# Engineering Smart Biomaterial Interfaces for iPSC-CM Maturation: A Biophysical and Metabolic Reprogramming Approach to Regenerative Cardiac Medicine

**DOI:** 10.3390/ijms27062637

**Published:** 2026-03-13

**Authors:** Dhienda C. Shahannaz, Tadahisa Sugiura

**Affiliations:** 1 Digestive Disease & Surgery Institute, Cleveland Clinic, Cleveland, OH 44195, USA; dhiendaladdynasrul@gmail.com; 2 Department of Cardiothoracic and Vascular Surgery, Montefiore Medical Center, Albert Einstein College of Medicine, Bronx, NY 10467, USA

**Keywords:** iPSC-CMs, cardiomyocyte maturation, smart biomaterials, biophysical cues, metabolic reprogramming, mechanotransduction, mitochondrial biogenesis, electroconductive scaffolds, topography, tissue engineering

## Abstract

The maturation of induced pluripotent stem cell-derived cardiomyocytes (iPSC-CMs) remains a major translational bottleneck in regenerative cardiac medicine, as current differentiation platforms yield electrophysiologically and metabolically immature phenotypes. This review explores emerging strategies to engineer “smart” biomaterial interfaces that actively instruct iPSC-CM maturation through synergistic biophysical and metabolic reprogramming. By integrating nanotopographical patterning, mechanoelectric coupling, and tunable substrate stiffness with metabolic interventions such as mitochondrial substrate optimization and fatty acid oxidation induction, the literature reveals consistent links between cell–matrix crosstalk, sarcomeric organization, calcium handling, and oxidative metabolism. Recent advances in bioactive scaffolds and extracellular vesicle (EV)-functionalized hydrogels are highlighted as platforms capable of approximating key features of the myocardium’s native electromechanical and bioenergetic environment. Across two- and three-dimensional culture systems, this review identifies recurring maturation patterns, persistent gaps in metric standardization and long-term phenotype stability, and ongoing limitations related to scalability and translational implementation. Collectively, the findings synthesized here indicate that convergence between biomaterial engineering and metabolic programming represents a critical design principle for advancing iPSC-CMs toward functionally mature, clinically relevant phenotypes. This integrated approach enhances the fidelity of iPSC-CMs for disease modeling, drug screening, and regenerative cardiac therapies.

## 1. Introduction

### 1.1. Context and Background

Human induced pluripotent stem cell-derived cardiomyocytes (iPSC-CMs) have emerged as a transformative platform for cardiac disease modeling, drug discovery, and regenerative therapy [1,2,3]. Their scalability, genetic tractability, and capacity for patient-specific modeling position them at the forefront of modern regenerative cardiac medicine. Despite substantial advances in differentiation protocols, however, iPSC-CMs consistently retain an immature phenotype. Hallmark features of this immaturity include fetal-like sarcomeric organization, underdeveloped excitation–contraction coupling, immature electrophysiological properties, and a predominantly glycolytic metabolic profile. Collectively, these limitations represent a major translational bottleneck, restricting both the predictive fidelity of in vitro models and the functional integration of iPSC-CMs in therapeutic settings [4,5,6,7].

In vivo, cardiomyocyte maturation unfolds within a highly structured and dynamic microenvironment in which extracellular matrix architecture, mechanical loading, electrical signaling, and metabolic availability co-evolve across developmental time. Recapitulating this multifactorial milieu in vitro has driven the emergence of smart biomaterial interfaces—engineered substrates and scaffolds designed not only to support cardiomyocyte attachment, but to actively instruct cellular behavior through defined physical and biochemical cues [6,7,8,9,10,11]. Advances in materials science now permit precise control over substrate stiffness, anisotropy, topography, and electrical conductivity, enabling increasingly faithful emulation of key aspects of the myocardial niche.

At the same time, cardiomyocyte maturation is increasingly recognized as a fundamentally bioenergetic process. Developmental progression from fetal to adult myocardium is accompanied by a metabolic transition from glycolytic reliance toward mitochondrial oxidative phosphorylation and fatty acid utilization. This metabolic reprogramming is tightly coupled to sarcomeric organization, calcium handling, and force generation, underscoring that structural, electrophysiological, and metabolic maturation are not independent outcomes but interdependent processes. Notably, biophysical stimuli delivered through biomaterial interfaces have been shown to directly influence mitochondrial biogenesis, dynamics, and substrate preference, positioning the cell–material interface as a critical regulatory nexus linking mechanics and metabolism.

### 1.2. Objectives of This Review

Despite rapid progress in interface design and maturation strategies, the field remains fragmented. Biomaterial engineering, mechanical stimulation, electrical conditioning, and metabolic modulation are frequently investigated in isolation, often using disparate platforms, outcome measures, and reporting standards. This fragmentation complicates cross-study comparison and obscures generalizable principles governing cardiomyocyte maturation. Consequently, a unified synthesis that integrates interface-mediated mechanotransduction with downstream metabolic and sarcomeric remodeling remains lacking.

A consolidated synthesis is therefore needed to clarify how smart biomaterial interfaces function as active regulators of cardiomyocyte fate, rather than passive supports, and how their physical properties converge with intracellular metabolic remodeling to drive functional maturation. Mapping this convergence is essential for advancing reproducible, physiologically relevant iPSC-CM platforms suitable for regenerative and precision medicine applications.

The objective of this review is to synthesize and comparatively analyze maturation strategies in which biomaterial interfaces function as active, instructive regulators of coupled biophysical and metabolic maturation in iPSC-derived cardiomyocytes. Emphasis is placed on elucidating how interface-driven biophysical cues—including substrate stiffness, topographical organization, dynamic mechanical loading, and electrical conductivity—integrate with metabolic reprogramming processes such as mitochondrial maturation and shifts in energy substrate utilization. By unifying perspectives from materials science, mechanobiology, and cardiac metabolism, this review seeks to establish a coherent conceptual framework to inform rational interface design and accelerate translational progress toward functionally mature cardiomyocyte platforms.

In this review, maturation is defined as the progression of iPSC-derived cardiomyocytes toward adult-like phenotypes across multiple, non-synchronous dimensions, including (i) structural maturation (sarcomeric alignment and cellular morphology), (ii) electrophysiological maturation (action potential characteristics and calcium handling), (iii) metabolic maturation (mitochondrial biogenesis, oxidative phosphorylation, and fatty acid utilization), and (iv) functional maturation (force generation and contractile efficiency). Importantly, most existing experimental systems achieve partial or intermediate maturation states rather than fully adult-equivalent phenotypes. This narrative review integrates structured evidence mapping to synthesize convergent biophysical–metabolic design principles rather than performing a formal systematic or scoping review.

## 2. Mapping the Landscape

This section surveys and comparatively synthesizes existing biomaterial-based strategies for iPSC-CM maturation, organized by dominant biophysical and metabolic design principles.

### 2.1. Biomaterial Categories and Design Rationales

Across the included studies, smart biomaterial interfaces were systematically categorized according to their dominant instructive features, including topographical organization, mechanical stiffness, electrical conductivity, dynamic mechanical loading, and culture dimensionality (2D versus 3D) [12,13,14]. Despite substantial heterogeneity in material composition and fabrication strategies, convergent relationships consistently emerged between specific interface properties and reproducible structural, electrophysiological, and metabolic maturation outcomes in iPSC-derived cardiomyocytes. Unless otherwise specified, maturation outcomes discussed here reflect relative improvements within each dimension rather than attainment of fully adult human cardiomyocyte benchmarks. These relationships, organized by biomaterial category and underlying design rationale, are comparatively summarized in Table 1.

Micro- and nano-patterned interfaces incorporating nanogrooves, ridges, aligned fibers, and anisotropic architectures consistently promoted elongated cardiomyocyte morphology, directional sarcomeric alignment, and increased conduction velocity across multiple material platforms (Table 1; Topographically Patterned Interfaces) [12,13,14]. Substrates designed to recapitulate myocardial fiber anisotropy reproducibly outperformed isotropic controls, reinforcing spatial guidance as a dominant determinant of structural maturation.

Mechanical stiffness emerged as a second central design axis. Across natural, synthetic, and hybrid biomaterials, substrates tuned to elastic moduli approximating native myocardium—typically within the 5–20 kPa range—were most consistently associated with organized sarcomeres, enhanced force generation, and coordinated contraction (Table 1; Stiffness-Tuned Substrates) [15,16,17,18]. In contrast, excessively rigid or overly compliant substrates were frequently linked to cytoskeletal disarray, reduced contractile performance, and immature electrophysiological behavior [18,19,20,21], underscoring the narrow mechanical window within which optimal maturation occurs. Compared with topographical patterning alone, stiffness-tuned substrates more reliably enhance contractile force generation but exert weaker effects on metabolic switching unless combined with electrical or biochemical cues [12,17,18].

When stratified by material class, natural biomaterials such as collagen, fibrin, and decellularized extracellular matrix primarily supported maturation through native biochemical ligands and adhesion motifs, but exhibited limited stiffness tunability and batch-to-batch variability (Table 1; Natural Biomaterials) [22,23,24]. Synthetic platforms—including polyacrylamide, PDMS, PEG-based hydrogels, and conductive polymers—enabled precise control over mechanical and electrical properties, yielding more reproducible maturation outcomes when appropriately biofunctionalized (Table 1; Synthetic Biomaterials) [24,25,26,27,28]. Hybrid systems combining natural and synthetic components consistently demonstrated the most robust and balanced maturation phenotypes, reflecting the advantage of decoupling biochemical signaling from mechanical control while preserving tunability (Table 1; Hybrid Biomaterials) [29,30,31,32,33].

Three-dimensional smart matrices and engineered cardiac tissues further enhanced structural organization, electrophysiological stability, and contractile coordination compared to two-dimensional systems (Table 1; 3D Smart Matrices). These improvements were frequently accompanied by adult-like metabolomic signatures and increased oxidative phosphorylation. However, such gains were offset by increased heterogeneity, diffusion constraints, and reduced experimental throughput, highlighting an inherent trade-off between physiological relevance and experimental control.

**Table 1:** Included studies summary.

Biomaterial Category	Representative Materials	Key Design Features	Primary Maturation Outcomes	Metabolic Effects	Key Limitations
Natural Biomaterials	Collagen, gelatin, fibrin, decellularized ECM (dECM)	Native biochemical ligands; inherent cell adhesion motifs; limited stiffness tunability	Improved cell attachment; enhanced sarcomeric organization; modest improvement in calcium handling	Partial shift toward oxidative metabolism; increased mitochondrial content but limited functional coupling	Batch variability; poor long-term mechanical stability; limited control over elastic modulus; difficult decoupling of biochemical vs. mechanical effects
Synthetic Biomaterials	Polacrylamide, PDMS, PEG hydrogels, conductive polymers	Precisely tunable stiffness (typically 1–50 kPa); controllable degradation; customizable electrical conductivity	Improved sarcomere alignment at physiological stiffness (5–20 kPa) [18,34,35]; enhanced force generation; improved conduction velocity	Increased mitochondrial biogenesis and fusion at physiological stiffness; enhanced respiratory capacity	Lack of intrinsic bioactivity; requires surface functionalization; reduced cell viability without biochemical cues
Hybrid Biomaterials	ECM-PEG composites, gelatin-methacrylate, ECM_coated PDMS	Decoupled biochemical and mechanical signaling, spatial and temporal tunability	Robust structural maturation; anisotropic alignment; improved electrophysiological stability	Consistent glycolytic-to-oxidative metabolic transition; enhanced FAO gene expression	Increased fabrication complexity; scalability challenges; batch-to-batch reproducibility concerns
Topographically Patterned Interfaces	Nanogrooves, aligned fibers, ridges, anisotropic microchannels	Spatial guidance cues mimicking myocardial fiber architecture	Elongated cell morphology; directional sarcomere alignment; increased conduction velocity	Indirect promotion of oxidative metabolism via cytoskeletal organization	Limited control over biochemical signaling; pattern fidelity degradation over long culture durations
Stiffness-Tuned Substrates	Tunable hydrogels, elastomers	Elastic modulus tuned to native myocardium (≈5–20 kPa)	Optimal contractile force generation; organized cytoskeleton; adult-like sarcomeric spacing	Enhanced mitochondrial biogenesis and fusion at physiological stiffness	Excessive rigidity or compliance disrupts maturation; narrow optimal mechanical window
Electrically Conductive Platforms	Carbon nanotubes graphene, conductive polymers	Enhanced electrical coupling; reduced excitation thresholds	Improved action potential propagation; synchronized contractions; enhanced calcium cycling	Increased mitochondrial efficiency secondary to improved excitation–contraction coupling	Potential cytotoxicity; long-term stability concerns; regulatory uncertainty
Dynamic Mechanical Systems	Cyclic strain platforms, stretchable scaffolds	Time-varying mechanical load; mimic cardiac strain	Increased contractile amplitude; adult isoform expression; improved force–frequency response	Reinforced oxidative metabolism via mechanoresponsive transcription	Technical complexity; limited standardization across platforms
3D Smart Matrices	Aligned hydrogels, engineered cardiac tissues	3D architecture; diffusion gradients; multicellular organization	Superior structural and functional maturation compared to 2D [36,37,38]; improved electrophysiological stability	Adult-like metabolomic profiles; increased oxidative phosphorylation	Increased heterogeneity; diffusion limitations; reduced experimental throughput

This table synthesizes representative studies by biomaterial category, highlighting dominant design features, primary structural and functional maturation outcomes, associated metabolic effects, and key experimental limitations. The table is intended as an integrative reference to facilitate cross-platform comparison rather than an exhaustive catalog of individual studies. Reported maturation outcomes reflect trends consistently observed across multiple publications within each category, acknowledging variability in culture duration, cell source, and analytical endpoints.

However, several studies report that prolonged electrical stimulation can induce electrophysiological instability or calcium overload in immature cells, highlighting a narrow therapeutic window for pacing-based maturation [39,40,41,42,43,44]. Collectively, the data summarized in Table 1 delineate a convergent maturation landscape in which anisotropic architecture, physiological stiffness, and controlled electrical or mechanical stimulation act as dominant drivers of structural maturation across diverse biomaterial platforms. Importantly, these structural features frequently co-occurred with metabolic and mitochondrial adaptations, providing the empirical foundation for the integrated biophysical–metabolic synthesis developed in subsequent sections.

While these comparative trends delineate which biomaterial features are most consistently associated with maturation outcomes, they do not explain how extracellular interface properties are translated into intracellular structural, electrophysiological, and metabolic changes. The following section therefore examines the mechanotransduction pathways through which biophysical cues exert their effects.

### 2.2. Biophysical Cues Driving Structural and Functional Maturation

Beyond static material properties, many biomaterial platforms incorporated dynamic biophysical cues to more closely recapitulate the cardiac microenvironment. Among these, electrical stimulation has been one of the most extensively explored modalities, with multiple studies reporting enhanced action potential propagation, increased conduction velocity, and improved calcium handling following chronic pacing [1,2,3]. Electrically conductive substrates, including carbon-based materials and conductive polymers [39,40,41,42,43,44], further facilitated synchronized contractions and reduced excitation thresholds.

Mechanical stimulation through cyclic strain and stretch was also widely reported to promote maturation [16,45,46,47]. Cyclic loading induced sarcomeric realignment along the principal strain axis, increased contractile force generation, and enhanced expression of adult isoforms of contractile proteins [16,44,45]. In parallel, shear stress—primarily investigated in microfluidic systems—contributed to improved electrophysiological stability and metabolic adaptation, particularly in three-dimensional constructs [16,48].

At the molecular level, these diverse biophysical cues converge on conserved mechanosensing and mechanotransduction pathways. Studies consistently implicate integrin–focal adhesion kinase (FAK) signaling, cytoskeletal remodeling, and downstream MAPK cascades as central mediators translating extracellular mechanical inputs into intracellular responses [49,50,51,52]. Several reports further identify the YAP/TAZ transcriptional regulators as key nodes linking substrate stiffness, mechanical strain, and topographical guidance to gene expression programs associated with both structural and metabolic maturation [53,54,55,56,57]. Cytoskeletal tension and actin organization emerge as common upstream regulators coordinating extracellular mechanics with intracellular signaling networks [49,53,58].

Notably, evidence regarding the sufficiency of mechanical stimulation alone remains mixed. While some studies report substantial improvements in sarcomeric alignment and force production under cyclic strain, others observe limited metabolic maturation in the absence of concurrent substrate optimization or hormonal modulation [45,46,47]. These findings underscore that biophysical cues rarely act in isolation.

Collectively, the literature indicates that biophysical stimulation functions as a coordinated regulatory system rather than a single trigger, shaping cardiomyocyte architecture, electrophysiology, and intracellular organization through integrated mechanotransductive networks. Unless otherwise specified, maturation outcomes discussed here reflect relative improvements within each functional dimension rather than attainment of fully adult human cardiomyocyte benchmarks. Table 2 summarizes representative biomaterial platforms incorporating dynamic biophysical cues—electrical, mechanical, and shear stimulation—and their reported effects on cardiomyocyte structural, electrophysiological, and metabolic maturation, along with the principal mechanotransductive pathways implicated.

**Figure 1:**
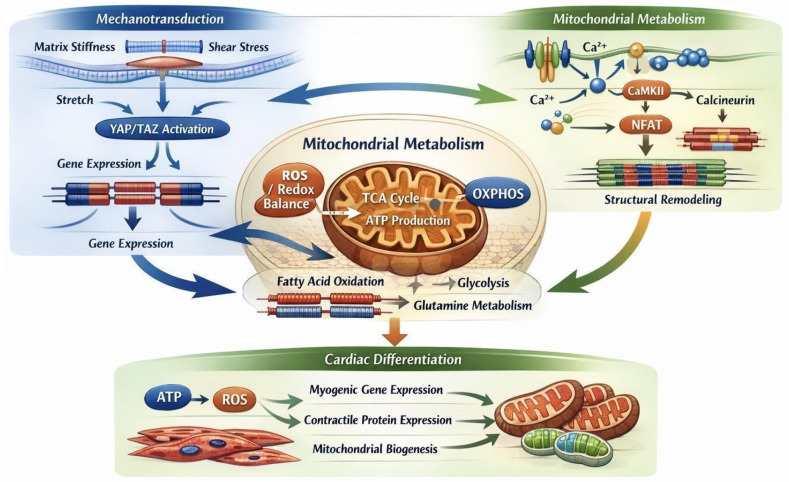
Multi-scale mechanotransduction–metabolic pathway map underlying iPSC-CM maturation on smart biomaterial interfaces. This schematic depicts the conceptual relationships between biophysical cues [58]—substrate stiffness, topographical alignment, cyclic strain, electrical stimulation, shear stress, and 3D confinement—and their effects on cardiomyocyte structural, electrophysiological, and metabolic maturation. Arrows indicate key signaling pathways linking extracellular inputs to intracellular responses, including integrin–FAK, RhoA–ROCK, calcium-dependent signaling, and YAP/TAZ transcriptional regulation. Downstream effects on mitochondrial dynamics (biogenesis, fusion), oxidative phosphorylation, and fatty acid metabolism are illustrated. Mechanistic details are provided for context but are not exhaustive. The schematic emphasizes conceptual relationships rather than predictive or fully detailed molecular mechanisms. The schematic emphasizes conceptual relationships rather than exhaustive molecular detail.

These pathways do not operate independently but form an integrated multi-scale network in which extracellular mechanics, cytoskeletal organization, and mitochondrial metabolism are tightly coupled (Figure 1).

### 2.3. Metabolic Reprogramming and Its Coupling to Biophysical Instruction

A defining feature across maturation-focused studies is the induction of a metabolic shift from glycolytic reliance toward oxidative phosphorylation, mirroring the developmental trajectory of postnatal cardiomyocytes [1,4]. Across diverse biomaterial platforms, iPSC-CMs cultured under maturation-promoting conditions exhibit increased mitochondrial content, elongated mitochondrial morphology, enhanced respiratory capacity, and upregulation of genes involved in fatty acid oxidation, tricarboxylic acid cycle activity, and electron transport chain function [4,58,61,62,63,64].

Importantly, these metabolic changes are rarely reported in isolation. Substrate stiffness consistently emerges as a critical determinant of metabolic state, with platforms tuned to physiological myocardial stiffness ranges promoting mitochondrial biogenesis and fusion, whereas non-physiological stiffness values are associated with fragmented mitochondrial networks and reduced oxidative capacity [16,17,18]. These observations suggest that mechanical context directly influences mitochondrial dynamics, potentially through cytoskeletal–mitochondrial coupling and mechanoresponsive transcriptional programs [4].

Several studies further demonstrate that direct metabolic modulation—through fatty acid-enriched coatings, oxygen-permeable matrices, or controlled-release systems delivering metabolic regulators such as PGC-1α activators or thyroid hormone analogs—can amplify oxidative metabolism and accelerate maturation markers when implemented within an appropriate biophysical environment [4]. However, metabolic interventions alone are consistently insufficient to induce comprehensive maturation [65,66], reinforcing the necessity of coordinated mechanical, electrical, and biochemical instruction. Metabolic outcomes associated with each biomaterial category are summarized comparatively in Table 1.

Several landmark studies have demonstrated that metabolic maturation of iPSC-derived cardiomyocytes can be achieved through biochemical or pharmacological interventions alone, including small-molecule modulation, fatty acid supplementation, and intracellular pathway targeting. In the present review, these studies are referenced for contextual comparison rather than treated as primary analytical units, as they primarily define metabolic endpoints without directly interrogating the role of biomaterial interfaces as active, instructive regulators of maturation.

Notably, multiple studies demonstrate that direct metabolic manipulation—such as substrate switching or fatty acid-driven acceleration of oxidative metabolism—can robustly enhance mitochondrial respiration and bioenergetic capacity in iPSC-derived cardiomyocytes, even in the absence of advanced structural organization [67]. However, these metabolic gains frequently fail to coincide with proportional improvements in sarcomeric alignment, excitation–contraction coupling, or force generation, underscoring that metabolic acceleration alone lacks sufficient contextual integration to drive holistic cardiomyocyte maturation. This principle is exemplified by Horikoshi et al. [68], who showed that fatty acid treatment alone promotes an adult-like oxidative metabolic profile, including increased mitochondrial respiration and reliance on fatty acid oxidation, without the incorporation of engineered biophysical cues. While this work establishes that metabolic reprogramming is achievable in isolation, the resulting phenotype remains insufficiently contextualized in the absence of concurrent structural and electromechanical instruction. Similarly, Hu et al. [69] demonstrated that inhibition of HIF1α and LDHA is sufficient to force a glycolytic-to-oxidative metabolic transition in human iPSC-derived cardiomyocytes, markedly increasing mitochondrial respiration and oxidative capacity; however, this intervention alone did not recapitulate coordinated sarcomeric, electrophysiological, or contractile maturation. Collectively, these studies highlight a critical distinction between metabolic acceleration and integrated cardiomyocyte maturation. Importantly, a causal link between mechanical work and metabolic maturation has been demonstrated even in systems lacking instructive biomaterial interfaces. Using engineered heart tissue constructs, Ulmer et al. [70] showed that increased contractile work alone was sufficient to drive mitochondrial maturation, enhance oxidative phosphorylation, and shift energy metabolism toward an adult-like profile in hiPSC-derived cardiomyocytes. In this context, mechanical load emerged endogenously through tissue-level contraction rather than being imposed by substrate stiffness or topographical cues, with the surrounding matrix functioning primarily as a permissive scaffold rather than an instructive interface. Although such platforms fall outside the scope of biomaterial-driven strategies reviewed here, these findings provide mechanistic support for the tight coupling between force generation and energy metabolism, reinforcing the rationale for smart biomaterial interfaces: by preconfiguring mechanical context at the cell–material boundary, interface design can accelerate or stabilize metabolic maturation states that would otherwise require prolonged tissue-level remodeling.

Across the literature, biophysical cues and metabolic maturation features frequently co-emerge rather than appearing as isolated or sequential phenomena. Platforms optimized for physiological stiffness, anisotropic alignment, dynamic mechanical loading, or electrical stimulation are consistently associated with concurrent improvements in sarcomeric organization, electrophysiological stability, and metabolic gene expression profiles. Specifically, studies report enhanced expression of genes governing mitochondrial function, oxidative phosphorylation, and fatty acid metabolism in iPSC-CMs cultured on mechanically instructive substrates.

Conversely, studies incorporating metabolic modulation often report parallel improvements in cytoskeletal organization, contractile force generation, and calcium handling, suggesting bidirectional reinforcement between metabolic state and structural maturation. These coupled responses are observed across natural, synthetic, and hybrid biomaterial systems, as well as across two-dimensional and three-dimensional culture formats.

Comparative analyses between 2D and 3D systems further support this coupled maturation paradigm. Three-dimensional constructs—particularly those employing aligned matrices and dynamic mechanical or electrical stimulation—are more frequently associated with adult-like metabolomic profiles, increased mitochondrial content, higher contractile amplitude, and improved calcium handling relative to 2D platforms. At the same time, several studies report increased variability, diffusion limitations, and spatial heterogeneity in thicker 3D constructs, underscoring trade-offs between physiological relevance and experimental control.

**Figure 2:**
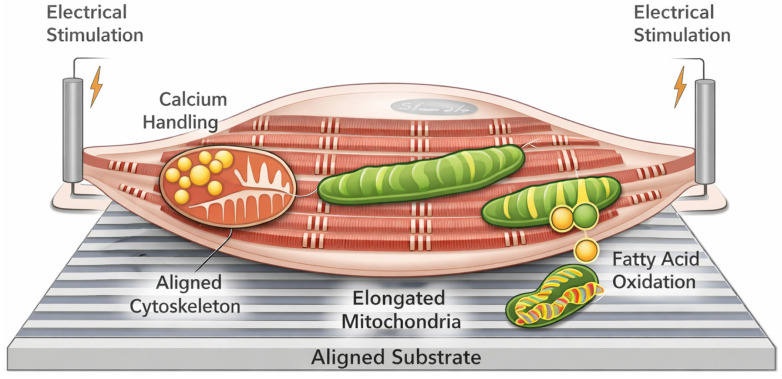
Conceptual schematic of iPSC-derived cardiomyocyte maturation responses to integrated biophysical and metabolic cues on smart biomaterial platforms. The figure depicts representative interactions between aligned and stiffness-tuned substrates, electrical stimulation or conductive materials, and cardiomyocyte structural, electrophysiological, and metabolic responses as described in existing studies. Shown pathways include cytoskeletal organization and focal adhesion formation, enhancement of excitation–contraction coupling and calcium handling, activation of integrin–FAK and YAP/TAZ-related signaling, and downstream mitochondrial adaptations such as elongation, increased oxidative phosphorylation, and fatty acid oxidation. Shaded regions and pathway groupings are derived from literature-based clustering of reported maturation phenotypes across two-dimensional, three-dimensional, and microengineered systems. No single study defines this schematic, and it is not intended to introduce a new mechanistic model. Instead, it visually organizes commonly reported maturation-associated features to support comparative interpretation of prior work.

Collectively, the reviewed studies indicate that biophysical interface properties and metabolic maturation markers tend to evolve in parallel under maturation-enhancing conditions, without consistently resolving a unidirectional causal hierarchy (Figure 2). Instead, effective maturation appears to arise from convergence between mechanical instruction and metabolic adaptation, mediated through shared signaling pathways and structural feedback mechanisms.

### 2.4. Emerging Technologies

Beyond established platforms, several emerging technologies are being developed to advance iPSC-CM maturation. Organ-on-chip systems combine engineered matrices with microfluidic control to precisely regulate mechanical, electrical, and metabolic parameters, providing reproducible maturation environments while maintaining physiologically relevant stimulation profiles. In these systems, the matrix properties—including stiffness, alignment, and topographical cues—directly influence cardiomyocyte organization and functional output.

Optogenetically responsive scaffolds offer spatiotemporally controlled stimulation through patterned light activation, enabling modulation of contractility without physical electrodes. The effectiveness of these scaffolds is closely linked to the underlying matrix, which transduces optical cues into cytoskeletal and contractile responses. Similarly, bioresponsive surfaces that dynamically adapt stiffness or conductivity in response to contractile forces introduce feedback-regulated maturation paradigms, in which matrix adaptation directly coordinates with cellular mechanical and metabolic states.

These approaches are discussed insofar as they illustrate future strategies for coordinated biophysical–metabolic control rather than as standalone solutions.

## 3. Discussion

This section critically interprets the synthesized evidence, identifies unresolved gaps, and contextualizes the proposed framework within translational constraints.

### 3.1. Integrative Maturity: Beyond One-Dimensional Approaches

The findings synthesized in this scoping review collectively reinforce a central conclusion: iPSC-CM maturation cannot be achieved through isolated interventions. Approaches that focus exclusively on biochemical supplementation, electrical pacing, or mechanical conditioning consistently yield partial phenotypes, improving select functional parameters while leaving others underdeveloped. This limitation is exemplified by studies in which metabolic pathways are directly reprogrammed—such as HIF1α/LDHA inhibition—yielding enhanced oxidative metabolism without full structural or functional integration [69]. This pattern persists even in studies reporting marked acceleration of oxidative metabolism through substrate reprogramming, where metabolic gains are not accompanied by equivalent structural or electrophysiological maturation [67]. In contrast, studies integrating smart biomaterial interfaces capable of delivering coordinated biophysical and metabolic instruction demonstrate more comprehensive and stable maturation outcomes.

The myocardium develops within a tightly regulated environment where mechanical load, electrical activity, and metabolic demand evolve in synchrony. Smart biomaterials that co-design substrate stiffness, topographical guidance, and electrical conductivity are reported to replicate this developmental logic by providing continuous feedback between the extracellular interface and intracellular energy systems. Substrate-mediated mechanotransduction influences cytoskeletal tension and nuclear signaling, which in turn regulates mitochondrial biogenesis (e.g., PGC-1α), substrate utilization, and oxidative capacity [4]. Collectively, these findings indicate that mechanical and metabolic maturation are not parallel processes but mutually reinforcing states.

Importantly, the reviewed literature indicates that metabolic reprogramming is rarely sustained in the absence of appropriate mechanical context. Supporting this limitation, Peters et al. [71] demonstrated that iPSC-derived cardiomyocytes driven toward oxidative metabolic maturation exhibited increased susceptibility to hypoxia-induced injury in the absence of concurrent biomechanical adaptation, underscoring that accelerated metabolic maturation alone may confer bioenergetic fragility rather than functional resilience. Conversely, mechanically optimized substrates fail to induce adult-like contractile energetics without concurrent metabolic adaptation. Based on recurring patterns reported across studies, the following section interprets their implications for interface-driven maturation. Notably, hybrid biomaterials (Table 1) are most consistently reported to support concurrent structural and metabolic maturation [14,22,29,31].

### 3.2. Critical Gaps

Despite rapid progress, several critical gaps persist across the field. One of the most significant limitations is the lack of standardized metrics for cardiomyocyte maturation. Studies employ heterogeneous endpoints—ranging from sarcomere length and calcium transient kinetics to transcriptomic signatures and mitochondrial morphology—making cross-platform comparisons difficult. Without consensus benchmarks [4], it remains challenging to define what constitutes functionally “mature” iPSC-CMs or to assess whether maturation achieved in vitro approximates adult human myocardium in a clinically meaningful way.

A second major gap lies in the limited understanding of long-term stability of induced maturation. Most studies evaluate maturation markers over relatively short culture periods, leaving unresolved whether induced phenotypes persist after withdrawal of stimulation or following implantation. This is particularly relevant for regenerative applications, where durability of functional maturity is essential for safety and efficacy. Longitudinal studies tracking structural, electrophysiological, and metabolic stability over extended time frames remain scarce.

Additionally, the field remains heavily reliant on rodent-derived extracellular matrix components or generic protein coatings that do not fully recapitulate the biochemical complexity of human cardiac tissue. While these materials offer experimental accessibility, they may introduce species-specific biases that limit translational relevance. The underrepresentation of humanized ECM formulations, including age-, disease-, or region-specific matrices, represents a critical opportunity for advancement.

Finally, scalability and reproducibility remain ongoing challenges. Many sophisticated biomaterial systems demonstrate impressive maturation effects but are difficult to standardize or manufacture at scale, limiting their applicability beyond proof-of-concept studies.

### 3.3. Translational Relevance and Constraints

From a translational perspective, several practical constraints must be considered alongside reported in vitro maturation outcomes. While several platforms demonstrate strong in vitro maturation effects, many remain unsuitable for clinical translation due to regulatory uncertainty, scalability limitations, or material reproducibility challenges.

Regulatory considerations include scaffold biocompatibility, degradation kinetics, and safety profiles, particularly for conductive nanomaterials such as carbon nanotubes or conductive polymers [39,41,42]. The long-term effects of such materials on host tissue and systemic toxicity remain incompletely characterized, creating hurdles for regulatory approval.

Manufacturing and batch variability represent another barrier. Complex hybrid hydrogels or organ-on-chip devices often rely on precise microfabrication or natural ECM extracts, which can introduce batch-to-batch variability and limit reproducibility [14,29,31]. Standardizing fabrication protocols and material characterization is essential for scaling from research to preclinical or clinical applications.

Research-only versus clinically plausible platforms: Many studies employ experimental matrices or electrical stimulation paradigms that are suitable for mechanistic discovery but are currently impractical for clinical translation [12,13,40]. Use of FDA-cleared or clinically validated biomaterials may accelerate regulatory approval but constrain design flexibility. Balancing innovation with translational feasibility will be critical as the field moves toward regenerative applications, disease modeling, and drug discovery [22,39,40].

### 3.4. Theoretical Framework Proposal: The Dual-Axis Maturation Matrix

**Figure 3:**
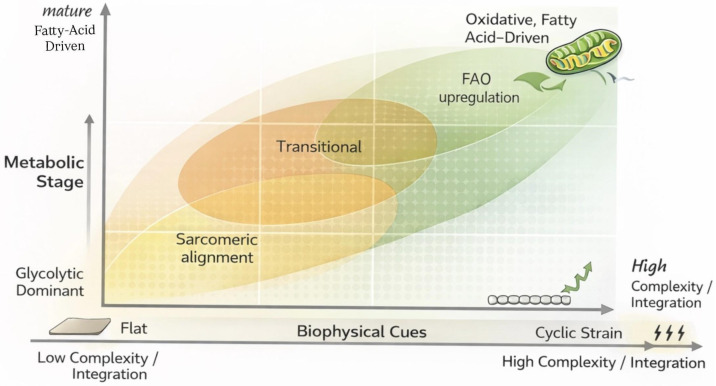
Dual-Axis Maturation Matrix for iPSC-CM Maturation on Smart Biomaterial Interfaces. Conceptual framework retrospectively organizing reported iPSC-derived cardiomyocyte (iPSC-CM) maturation outcomes across biomaterial platforms along two orthogonal dimensions. The X-axis represents increasing levels of biophysical instruction, encompassing substrate topography (flat to anisotropically aligned), elastic modulus (non-physiological to myocardial-relevant stiffness), mechanical loading (static to cyclic strain), and electrical stimulation (absent to chronic pacing or conductive substrates). The Y-axis represents metabolic stage, ranging from glycolytic-dominant phenotypes through transitional states to oxidative, fatty acid-driven metabolism. Shaded regions within the matrix indicate clusters of maturation phenotypes derived from patterns reported across multiple independent studies, rather than outcomes defined by any single experiment. The matrix does not represent a predictive or prescriptive model, but a post hoc organizational tool synthesizing heterogeneous maturation data across biomaterial systems. Its purpose is to facilitate comparison between platforms, clarify relationships between structural and metabolic maturation, and highlight underexplored combinations of biophysical and metabolic cues reported in the literature. Representative platforms from the literature are positioned illustratively within the matrix to demonstrate relative clustering rather than exact quantitative placement. Practical use: In practice, this framework may be used to benchmark emerging platforms against existing strategies, identify underexplored cue combinations, and guide experimental design toward coordinated rather than incremental maturation.

Based on recurring structural–metabolic maturation patterns reported across multiple biomaterial studies, and to address the fragmentation and heterogeneity in maturation strategies and outcome metrics documented in the preceding sections, we retrospectively organized reported findings into a Dual-Axis Maturation Matrix. In this framework, studies are mapped along two orthogonal dimensions (Figure 3):

- X-axis: Biophysical cues commonly explored in biomaterial-based maturation strategies include substrate stiffness [15,72], topographical alignment [44,73], mechanical loading [47,74], and electrical stimulation [62,75].
- Y-axis: Metabolic maturation is characterized by a transition from glycolytic dominance toward oxidative, fatty acid-driven metabolism, reflecting postnatal cardiomyocyte development [76,77,78]. Similar metabolic remodeling has been reported in iPSC-CMs through substrate manipulation, hormonal cues, and culture maturation strategies [17,79].

Explicit examples: For instance, anisotropically aligned hydrogels with physiological stiffness but without metabolic supplementation cluster in regions associated with advanced structural but incomplete metabolic maturation, whereas platforms combining electrical pacing with fatty acid supplementation occupy regions reflecting more coordinated maturation. Similarly, hybrid ECM-hydrogel scaffolds with integrated conductive polymers and hormonal cues demonstrate enhanced alignment between structural and energetic maturation states [12,13,29,40].

By positioning each biomaterial-based strategy within this matrix, researchers can more clearly evaluate how specific interface designs influence both structural and metabolic maturation; for example, platforms that optimize stiffness and alignment but fail to induce metabolic switching occupy a different region than systems integrating electrical pacing with fatty acid supplementation and mitochondrial maturation.

#### Limitations of the Dual-Axis Framework

The Dual-Axis Maturation Matrix is constrained by heterogeneity in maturation metrics, experimental durations, and metabolic assessment methods across studies. In particular, the absence of standardized benchmarks for defining metabolic state and functional maturity limits direct quantitative comparisons between platforms. Consequently, the matrix should be interpreted as a comparative synthesis of reported phenotypes rather than a definitive classification or predictive model. As the field progresses toward standardized metabolic and functional readouts, this framework may be refined to improve resolution and consistency.

## 4. Future Directions

The evolution of iPSC-CM maturation strategies increasingly points toward precision interface engineering, where biomaterials are no longer static design outputs but adaptive systems informed by biological feedback and data-driven optimization. Recent advances suggest that future progress will depend on three converging trajectories: intelligent material design, genetic and epigenetic integration, and high-resolution multi-omics validation.

### 4.1. Precision Biomaterial Engineering

One of the most promising directions is the application of computational and artificial intelligence-assisted design to biomaterial interfaces. Machine learning approaches are beginning to be used to predict how combinations of stiffness, anisotropy, conductivity, and topographical features influence cardiomyocyte behavior. Rather than iteratively testing single parameters, AI-guided platforms can explore high-dimensional design spaces, accelerating the identification of interface architectures that promote synchronized structural, electrophysiological, and metabolic maturation.

At the material level, nano-scale stiffness gradients and hierarchical topographies represent a critical next step. Native myocardium is mechanically heterogeneous, with regional variations in stiffness and fiber orientation that evolve during development and disease. Biomaterials capable of presenting spatially graded mechanical cues may better recapitulate these conditions, guiding cytoskeletal organization and force transmission in a developmentally relevant manner. Early evidence suggests that cardiomyocytes can sense and respond to subtle mechanical gradients, adjusting sarcomeric alignment and mitochondrial organization accordingly.

Dynamic and adaptive materials also represent an important frontier. Bioresponsive scaffolds that stiffen, soften, or alter conductivity in response to contractile force or electrical activity could establish feedback loops that reinforce maturation. Such systems move beyond static mimicry toward developmentally inspired reciprocity, where the interface evolves alongside the cell.

### 4.2. Integration with Genetic and Epigenetic Modulation

While biomaterials provide external instruction, genetic and epigenetic modulation offers an opportunity to align intracellular programs with interface-driven cues. Increasing attention has been given to the controlled manipulation of pathways governing mitochondrial biogenesis and mechanotransduction. Targeted upregulation of key regulators (e.g., PGC-1α for mitochondria, YAP/TAZ for mechanosensing) has shown potential to amplify maturation effects when combined with appropriate physical environments.

CRISPR-based tools enable precise, tunable, and potentially reversible control of these pathways. Future strategies are likely to favor transient or inducible modulation rather than permanent genetic alteration, minimizing safety concerns while enhancing functional outcomes. When paired with smart biomaterials, genetic modulation can be spatially or temporally coordinated, reinforcing the coupling between external mechanics and internal metabolism.

Epigenetic regulation is another underexplored dimension. Mechanical and metabolic cues are increasingly recognized as drivers of chromatin remodeling and epigenetic memory. Understanding how interface design influences epigenetic stability may be key to achieving long-lasting maturation, particularly where cells must retain their phenotype after implantation or prolonged culture.

### 4.3. Multi-Omics Profiling and Systems-Level Validation

As maturation strategies grow more complex, multi-omics profiling will be essential for rigorous validation. Transcriptomic analyses alone are insufficient to capture functional maturity, particularly given post-transcriptional regulation and metabolic control points. Integrating proteomic and metabolomic data provides a more faithful representation of cardiomyocyte state, revealing whether structural and electrophysiological improvements are supported by appropriate energy production and substrate utilization.

Recent studies employing combined transcriptomic–proteomic–metabolomic pipelines demonstrate that maturation is best understood as a systems-level convergence, rather than a single pathway shift. Future work should prioritize longitudinal, multi-omics profiling to assess not only the induction of maturity but its stability over time. These datasets also provide valuable training material for predictive models linking interface properties to cellular outcomes.

Standardization will be critical. Shared reference datasets, consensus marker panels, and interoperable analytical pipelines will enable meaningful comparison across laboratories and platforms. Without such harmonization, the field risks generating sophisticated but siloed solutions that are difficult to translate or reproduce.

### 4.4. Towards Clinically Relevant Platforms

Ultimately, future directions must remain anchored in translational feasibility. Biomaterials selected for maturation platforms should increasingly align with clinically approved or approvable materials, balancing innovation with regulatory realism. Scalable manufacturing, batch consistency, and integration with existing bioprocessing pipelines will determine whether advanced maturation strategies can move beyond the laboratory.

The convergence of intelligent materials, genetic modulation, and multi-omics validation positions the field to transition from proof-of-concept maturation toward predictable, reproducible, and clinically meaningful cardiomyocyte engineering.

## 5. Conclusions

The body of evidence synthesized in this review supports a fundamental redefinition of how iPSC-CM maturation is approached. Smart biomaterial interfaces are not passive scaffolds, but active regulators of cardiomyocyte fate, capable of orchestrating structural organization, electrophysiological function, and metabolic identity through coordinated biophysical and biochemical instruction.

A central conclusion emerging from recent work is that maturation is an integrative process. Mechanical cues, electrical stimulation, and metabolic programming do not operate independently; rather, they form a tightly coupled network in which extracellular interface design directly influences intracellular energy systems, and metabolic state feeds back to stabilize structural and functional phenotypes. Attempts to mature iPSC-CMs through isolated interventions consistently fall short of reproducing adult-like properties.

The dual strategy highlighted in this review—integrating mechanotransduction with bioenergetic reprogramming—offers a coherent framework for overcoming this limitation. By aligning substrate stiffness, topography, and dynamic stimulation with metabolic transitions toward oxidative phosphorylation, smart biomaterials enable maturation trajectories that are more physiologically faithful and functionally robust.

However, progress toward translation will require addressing several persistent challenges. The absence of standardized maturation metrics complicates cross-study comparison and obscures true functional equivalence to adult human cardiomyocytes. Long-term stability of induced maturity remains insufficiently characterized, particularly in contexts relevant to implantation or chronic drug exposure. Furthermore, the continued reliance on non-human or overly simplified extracellular matrices underscores the need for humanized, bioinspired material design.

Despite these challenges, the trajectory of the field is clear. Advances in precision material engineering, integration with genetic and epigenetic modulation, and systems-level multi-omics validation are converging toward a new generation of maturation platforms. These platforms are increasingly capable of producing iPSC-CMs that are not only differentiated, but structurally organized, metabolically competent, and functionally reliable.

In the context of regenerative cardiac medicine, this shift has profound implications. Mature iPSC-CMs supported by intelligent interfaces offer enhanced predictive power for disease modeling, improved fidelity for drug screening, and greater safety and efficacy potential for therapeutic applications. By reframing biomaterials as active participants in cellular programming, rather than inert supports, the field moves closer to realizing the full promise of stem cell-based cardiac regeneration.

## Figures and Tables

**Table 2:** Mechanotransduction pathways involved in iPSC-CM response to smart interfaces.

Biophysical Cue	Primary Sensors	Core Signaling Nodes	Downstream Transcriptional Regulators	Mitochondrial & Metabolic Outcomes	Maturation Phenotypes
Substrate stiffness (physiological range: ~5–20 kPa)	Integrins (β1, β3), focal adhesions	FAK–Src activation; RhoA–ROCK signaling	YAP/TAZ nuclear localization [54,59,60]; MRTF-A	Increased mitochondrial biogenesis; enhanced fusion (MFN1/2); elevated oxidative phosphorylation	Improved sarcomeric alignment; increased contractile force; adult-like calcium handling
Topographical alignment (nanogrooves, fibers)	Integrin clustering; actin stress fibers	Cytoskeletal tension; FAK–MAPK cascade	SRF–MRTF axis; YAP/TAZ [54,59,60]	Indirect promotion of oxidative metabolism via cytoskeletal stabilization	Elongated morphology; anisotropic sarcomere organization; increased conduction velocity
Cyclic mechanical strain	Mechanosensitive ion channels; integrin–cytoskeleton complexes	MAPK (ERK1/2); RhoA–ROCK	YAP/TAZ [54,59,60]; MEF2	Enhanced mitochondrial respiratory capacity; improved ATP production	Increased force generation; expression of adult contractile isoforms
Electrical stimulation/conductive substrates	Voltage-gated ion channels; electroconductive matrices	Ca^2+^-dependent signaling; CaMKII	PGC-1α; ERRα	Enhanced mitochondrial oxidative capacity; improved FAO gene expression	Synchronized contractions; improved excitation–contraction coupling
Shear stress (microfluidic systems)	Mechanosensitive channels; glycocalyx	PI3K–Akt; MAPK	NRF1/2	Mitochondrial efficiency optimization; metabolic flexibility	Electrophysiological stability; improved calcium cycling
3D matrix confinement	Cell–cell junctions; integrin networks	Hippo pathway modulation; FAK–YAP cross-talk	YAP/TAZ [54,59,60]; TEAD	Sustained mitochondrial maturation; oxidative metabolic dominance	Adult-like metabolomic profile; enhanced contractile amplitude

Schematic representation of iPSC-derived cardiomyocyte maturation on a smart biomaterial interface integrating biophysical and metabolic stimulation. Aligned and stiffness-tuned substrates provide spatial and mechanical cues that promote cytoskeletal organization and focal adhesion formation. Electrical stimulation and conductive matrices enhance excitation–contraction coupling and calcium handling. These biophysical inputs converge on mechanotransduction pathways involving integrin–FAK signaling and YAP/TAZ transcriptional regulation, which interface with mitochondrial biogenesis and metabolic reprogramming. The coordinated activation of these pathways drives mitochondrial elongation, increased fatty acid oxidation, and enhanced oxidative phosphorylation, resulting in improved sarcomeric alignment, contractile force generation, and electrophysiological maturation toward an adult-like cardiomyocyte phenotype.

## Data Availability

No new data were created or analyzed in this study. Data sharing is not applicable to this article.
